# Strategies of Environmental Adaptation in the Haloarchaeal Genera *Haloarcula* and *Natrinema*

**DOI:** 10.3390/microorganisms13040761

**Published:** 2025-03-27

**Authors:** Dáša Straková, Cristina Sánchez-Porro, Rafael R. de la Haba, Antonio Ventosa

**Affiliations:** Department of Microbiology and Parasitology, Faculty of Pharmacy, University of Sevilla, 41012 Sevilla, Spain; daska.strakova@gmail.com (D.S.); sanpor@us.es (C.S.-P.)

**Keywords:** environmental adaptation, haloarchaea, *Haloarcula*, heavy metal tolerance, hypersaline environments, *Natrinema*, thiamine biosynthesis

## Abstract

Haloarchaea, a group of extremophilic archaea, thrive in hypersaline environments characterized not only by high salinity but also by other extreme conditions, such as intense UV radiation, high osmotic pressure, heavy metal contamination, oxidative stress, and fluctuating temperatures. This study investigates the environmental adaptation strategies of species of two genera, *Haloarcula* and *Natrinema*, the second and third largest haloarchaeal genera, respectively, after *Halorubrum*. Comparative genomic analyses were conducted on 48 species from both genera to elucidate their genomic diversity, metabolic potential, and stress-tolerance mechanisms. The genomes revealed diverse metabolic pathways, including rhodopsin-mediated phototrophy, nitrogen assimilation, and thiamine biosynthesis, which support their survival and adaptation to extreme conditions. The analysis identified mechanisms for oxidative stress mitigation, DNA repair, “salt-in” and “salt-out” osmoregulatory strategies, adaptations to temperature shifts and heavy metal exposure, and immune defense. Experimental validation of four representative species, *Haloarcula terrestris* S1AR25-5A^T^, *Haloarcula saliterrae* S1CR25-12^T^, *Haloarcula onubensis* S3CR25-11^T^, and *Natrinema salsiterrestre* S1CR25-10^T^, isolated from the heavy-metal-rich hypersaline soils in the Odiel Saltmarshes (Huelva, Spain), demonstrated their tolerance, especially to arsenic, corroborating genomic predictions. This study advances our understanding of the resilience of haloarchaea under poly-extreme conditions and underscores their ecological significance and promise for biotechnological applications, such as the bioremediation of heavy-metal-polluted environments and the production of valuable biomolecules.

## 1. Introduction

Haloarchaea, members of the class *Halobacteria* within the domain *Archaea*, are microorganisms thriving at the limits of life’s extreme conditions. These extremophiles inhabit hypersaline environments, such as salt flats, saline lakes, and salt mines, which are characterized by high salinity, intense solar radiation, fluctuating temperatures, oxygen availability, and toxic compounds [1,2]. These harsh conditions pose significant challenges to microbial survival, yet haloarchaea have evolved a range of unique adaptations that enable them to endure and prosper. Predominantly aerobic and chemoorganotrophic haloarchaea derive energy from the degradation of organic compounds [3]. However, they also exhibit metabolic versatility, using light energy through rhodopsin-based phototrophy or performing anaerobic respiration using alternative electron acceptors such as nitrate [4,5]. Their ecological roles extend beyond hypersaline habitats, as they contribute to biogeochemical cycles, nutrient turnover, and ecosystem stability [6,7]. Resilience of haloarchaea to environmental stressors, including heavy metal toxicity, oxidative stress, and UV radiation, has positioned them as valuable candidates for biotechnological applications, such as bioremediation and bioplastics production [8,9,10,11].

Among haloarchaea, the genus *Halorubrum* is the largest, encompassing 40 species widely distributed across hypersaline environments [12]. Following *Halorubrum*, *Haloarcula* and *Natrinema* are the second and third largest genera within the class *Halobacteria* and are the focus of this study. Both genera exhibit diversity at multiple levels: species, genetic, metabolic, and ecological. The genus *Haloarcula*, belonging to the family *Haloarculaceae*, order *Halobacteriales*, was established in 1986 when *Halobacterium vallismortis* was reclassified as *Haloarcula vallismortis* [13]. Recent phylogenomic study [14] merged the genera *Haloarcula* and *Halomicroarcula* into a single genus, *Haloarcula*, to better reflect evolutionary relationships. Currently, the genus comprises 29 species; however, recent revisions proposed synonymizing three species based on genomic evidence [15,16]. The genus *Natrinema*, part of the family *Natrialbaceae*, order *Halobacteriales*, was described in 1998 by reclassifying two strains previously assigned to *Halobacterium* [17]. However, almost simultaneously, another related genus, *Haloterrigena*, was proposed [18]. Taxonomic ambiguity between *Natrinema* and *Haloterrigena* was recently resolved through comparative genomic studies, leading to the reclassification of several *Haloterrigena* species into *Natrinema* [19]. With 22 recognized species, *Natrinema* demonstrates ecological and phylogenetic diversity, inhabiting hypersaline environments such as salt lakes, salterns, and hypersaline soils.

Despite their adaptability, the mechanisms underlying haloarchaeal responses to extreme environmental stressors, such as high salinity, heavy metal exposure, and oxidative stress, remain areas of active research. The growing impacts of climate change, combined with pollution from industrialization, are contributing to elevated salinity levels mainly in water systems [8]. Harnessing the bioremediation potential of halophilic microorganisms presents a viable and innovative approach to addressing these challenges. The objective of this study was to investigate the adaptive mechanisms of two haloarchaeal genera, with a particular focus on their heavy metal tolerance. By experimental analysis of four representative species isolated from the hypersaline soils of the Odiel Saltmarshes—a habitat characterized by high salinity and elevated heavy metal concentrations—the research aimed to determine their ability to cope with heavy metal stress, particularly with arsenic. Additionally, this study was conducted to explore the metabolic functions, osmoregulatory strategies, and other environmental adaptations that enable these haloarchaea to thrive under poly-extreme conditions. Furthermore, the objective was to examine the potential for *de novo* thiamine biosynthesis in *Haloarcula* and *Natrinema* species, providing insights into their metabolic complexity.

## 2. Materials and Methods

### 2.1. Genomic and Metabolic Profiling

A total of 48 genomic sequences of type strains, comprising 26 from *Haloarcula* species and 22 from *Natrinema* species, were retrieved from the NCBI GenBank database for analysis. The accession numbers of the genomes used are provided in Supplementary Table S1. The pangenome analysis was performed using the Enveomics toolkit v.1.0 [20]. To visualize core, variable (“shell”), and strain-specific genes, a flower plot was generated using the SRplot server [21], with the variable genes manually integrated into the visualization. Graphical depictions illustrating the evolution of both the pan-genomes and core-genomes for the genera *Haloarcula* and *Natrinema* were generated using the Pan-Genome Profile Analyze Tool (PanGP) v.1.0.1, following the recommended algorithms [22]. Functional annotation of orthologous genes was carried out using the BlastKOALA tool v.3.0 [23], assigning KEGG Orthology (KO) numbers and mapping them to KEGG pathways and modules for reconstructing metabolic pathways. In addition, CRISPR-Cas systems were identified using the CRISPRCasFinder tool v. 4.2.21 [24]. The amino acid frequency and isoelectric points were determined from reading frames translated into protein sequences. The isoelectric points of predicted proteomes were computed with the “iep” tool from the EMBOSS package v.6.5.7.0 [25] and visualized with the R package “ggplot2” v.3.5.1 [26]. Amino acid frequencies were analyzed with the “Biostrings” v.2.74.1 package in R [27], and a radar (spider) plot representing the amino acid frequencies was generated using the R package “fmsb” v.0.7.6 [28].

### 2.2. Assessment of Heavy Metal Tolerance

The distribution of genes associated with arsenic tolerance within the genera *Haloarcula* and *Natrinema* was highlighted using approximately maximum-likelihood phylogenomic trees. They were reconstructed using FastTreeMP v.2.1.8 [29] based on 1599 and 1503 core-orthologous protein sequences, respectively. Branch support values (%) were computed with the Shimodaira–Hasegawa test.

The susceptibility of four selected representatives (*Haloarcula terrestris* S1AR25-5A^T^, *Haloarcula saliterrae* S1CR25-12^T^, *Haloarcula onubensis* S3CR25-11^T^, and *Natrinema salsiterrestre* S1CR25-10^T^), isolated from hypersaline soils in the Odiel Saltmarshes, located in Huelva, Southwest Spain, to five heavy metals (arsenic, cadmium, copper, lead, and zinc) was assessed. Cultures of these strains—incubated for seven days in Reasoner’s 2A (R2A) broth medium (Difco, Franklin Lakes, NJ, USA) supplemented with a 25% (*w*/*v*) seawater salt solution, prepared by diluting a 30% (*w*/*v*) stock solution following Subov [30]—were used to inoculate Petri dishes containing R2A 25% (*w*/*v*) agar medium. The seawater salt solution consisted of (g/L): NaCl, 195; MgCl_2_ · 6H_2_O, 32.5; MgSO_4_ · 7H_2_O, 50.8; CaCl_2_, 0.83; KCl, 5.0; NaHCO_3_, 0.17; and NaBr, 0.58. The agar medium was supplemented with varying concentrations of heavy metal salts, including C_2_H_6_AsNaO_2_ · 3H_2_O, CdCl_2_ · H_2_O, CuSO_4_, Pb(C_2_H_3_O_2_)_2_ · 3H_2_O, and ZnSO_4_ · 7H_2_O. Concentrations ranged from 0.01 mM to 700 mM (0.01, 0.05, 0.5, 1, 2.5, 4, 5, 10, 20, 50, 80, 100, 150, 200, 300, 500, 600, and 700 mM, respectively) [31]. The heavy metal solutions were sterilized through 0.2 μm filter membranes before being added to the medium. The tolerance assays were conducted in duplicates. Following incubation at 37 °C for 1 to 4 weeks, colony growth in media with each heavy metal was evaluated. The lowest concentration of each heavy metal that completely inhibited haloarchaeal growth was recorded as the minimum inhibitory concentration (MIC). R2A 25% (*w*/*v*) agar media without heavy metals were used as controls for each isolate.

## 3. Results and Discussion

### 3.1. Comparative Genomic Analysis and Pan-Genome Dynamics of the Genera Haloarcula and Natrinema

**Figure 1:**
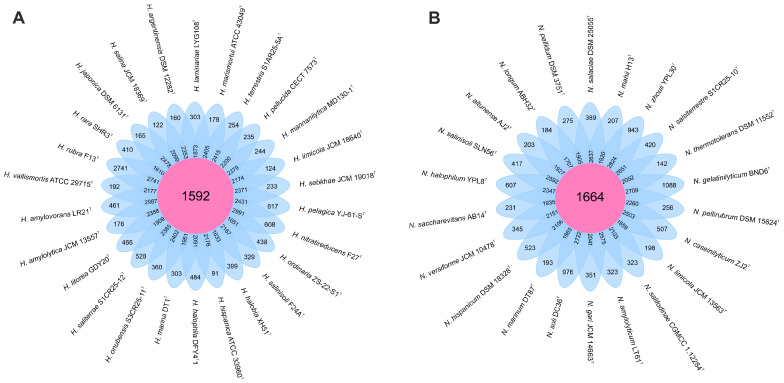
Flower plot showing the core (in the center), variable (in the annulus), and strain-specific (in the petals) genes of (**A**) the 26 *Haloarcula* species and (**B**) the 22 *Natrinema* species.

We analyzed 26 genomes from species of the genus *Haloarcula* and 22 genomes from those of the genus *Natrinema*. Within the *Haloarcula* genomes, a total of 108,027 translated coding sequences (CDSs) were identified and categorized into 8835 orthologous gene clusters (OGs), comprising 1592 core OGs and 7243 variable OGs. Additionally, 8198 singleton gene clusters were detected, resulting in a pan-genome encompassing 17,033 distinct gene clusters. In the *Natrinema* genomes, 94,900 protein CDSs were identified and grouped into 8235 orthologous gene clusters, including 1664 core OGs and 6571 variable OGs. Furthermore, 9101 singleton gene clusters were identified, contributing to a pan-genome comprising 17,336 different gene clusters. The flower plots in Figure 1 illustrate the distribution of core, variable (shared by multiple but not all strains), and singleton (strain-specific) gene clusters across the species within the genera *Haloarcula* and *Natrinema*.

**Figure 2:**
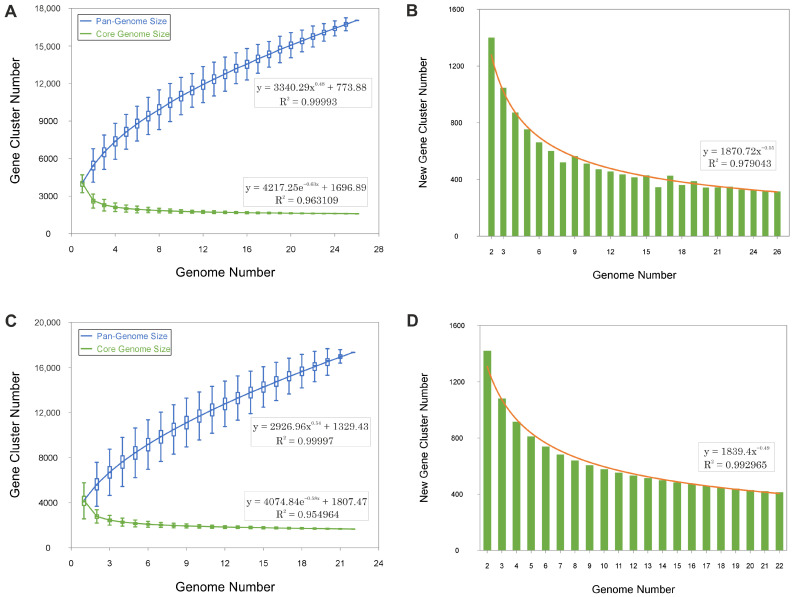
Gene accumulation curves of the pan-genomes (blue) and core-genomes (green) for species of the genera *Haloarcula* (**A**,**B**) and *Natrinema* (**C**,**D**). Panels A and C display the empirical gene accumulation curves, while panels B and D illustrate the least squares fit of the power law applied to the average values. The power law fit parameters and their respective coefficients of determination (R^2^) are shown for each dataset.

The pan-genomes of *Haloarcula* and *Natrinema* exhibit continuous expansions with each additional genome, as demonstrated by the steep upward trend in the pan-genome size curves (Figure 2A,C). The ongoing identification of novel genes (Figure 2B,D) further supports this pattern. This indicates that both genera possess an open pan-genome, wherein the discovery of new genes persists as additional genomes are analyzed, reflecting a high degree of genetic diversity and a dynamic capacity for gene acquisition.

### 3.2. Metabolic Potential and Pathway Analysis

#### 3.2.1. Carbohydrate Metabolism

The primary metabolic pathways of *Haloarcula* and *Natrinema* species were elucidated through genome analysis and functional annotation using the BlastKOALA tool. The species of both genera have key carbohydrate metabolism pathways, including gluconeogenesis and the semi-phosphorylative Entner–Doudoroff glycolytic pathway, enabling the conversion of glucose and other hexoses into pyruvate for entry into the tricarboxylic acid cycle. The Embden–Meyerhof–Parnas glycolytic pathway was absent in most species, with the exception of four *Natrinema* species (Supplementary Table S2). In addition, the species of both genera exhibit metabolic versatility, utilizing alternative carbon sources, like glycerol. Glycerol metabolism can proceed through phosphorylation by glycerol kinase to form *sn*-glycerol-3-phosphate (G3P), followed by its oxidation to dihydroxyacetone phosphate (DHAP) via G3P dehydrogenase, or through oxidation by glycerol dehydrogenase to dihydroxyacetone (DHA), which is subsequently phosphorylated by DHA kinase to form DHAP [32]. The G3P pathway was present in all *Haloarcula* and *Natrinema* species, except for *Natrinema salifodinaeae* CGMCC 1.12284^T^ (Supplementary Table S2). Furthermore, a fructose-specific phosphoenolpyruvate (PEP)-dependent phosphotransferase system (PTS) was found in most *Haloarcula* species and a few *Natrinema* species, facilitating fructose uptake and metabolism [33,34]. Moreover, *gat* genes involved in galactitol (dulcitol) metabolism as part of the bacterial-type PTS system [35] were identified in *Haloarcula amylovorans* LR21^T^ (Supplementary Table S2). Both genera also harbored genes for synthesizing polyhydroxyalkanoates (PHAs), biopolymers produced during nutrient-limited conditions that serve as carbon storage, offering potential for sustainable plastic alternatives [36,37,38,39,40].

#### 3.2.2. Nitrogen Metabolism

Genes encoding glutamine synthetase, glutamate synthase, and glutamate dehydrogenase, which are involved in ammonia assimilation [41], were identified in all genomes. Additionally, urease gene clusters (*ure*ABC and *ure*DEFGH), responsible for urea hydrolysis into ammonia and CO_2_ [42], were present in most *Haloarcula* species and a few *Natrinema* species. Some species possessed assimilatory nitrate and nitrite reductases, enabling nitrate utilization when ammonia or urea are scarce, enhancing adaptability in nitrogen-fluctuating environments. Furthermore, the majority of *Natrinema* species and half of the *Haloarcula* species encoded nitroalkane oxidase, catalyzing nitroalkanes to nitrite [43], which can be further reduced to ammonia. Nitrilase was identified in some *Haloarcula* species and *Natrinema zhouii* YPL30^T^, while formamidase and cyanate lyase were found in specific *Haloarcula* species (Supplementary Table S2), reflecting metabolic flexibility in utilizing various nitrogen sources and detoxifying harmful compounds.

#### 3.2.3. Transporters

The metabolic versatility is also supported by a variety of transporters. Multiple ABC transporters for sugars, phosphates, amino acids, and metals were prevalent across both genera (Supplementary Table S2). Transport systems for glucose/mannose and arabinogalactan oligomers were particularly abundant in *Haloarcula*. Ammonia transporters were detected across all genomes. A urea transporter (UrtABCDE) was identified in *Haloarcula* species containing urease genes, although only three *Natrinema* species encoded an active urea transport system. *Natrinema* species containing urease but lacking the *urtABCDE* gene cluster may have adapted to environments with high urea concentrations, allowing urea to enter the cell through passive diffusion [44]. Phosphate ABC transporters were present across all genomes, often appearing in multiple copies. Approximately half of the *Natrinema* and *Haloarcula* genomes also contained genes encoding a phosphonate ABC transporter. The TupABC tungstate transport system was present in all *Natrinema* species except for *Natrinema marinum* DT87^T^. No *Haloarcula* species originally classified under *Halomicroarcula* and later reassigned to *Haloarcula* possessed an ABC tungstate transport system (Supplementary Table S2). This system is crucial for the high-affinity uptake of tungstate ions (WO_4_^2−^), which are essential for tungsten-dependent enzymes, such as aldehyde ferredoxin oxidoreductase and formate dehydrogenase, involved in anaerobic metabolism and energy conservation [45]. Additionally, zinc ABC transporters were detected in all *Natrinema* genomes and in most *Haloarcula* genomes. In contrast, the CbiMNQO cobalt/nickel transport system was less common, found in some *Haloarcula* species and only two *Natrinema* species (Supplementary Table S2).

#### 3.2.4. Phototrophy

**Figure 3:**
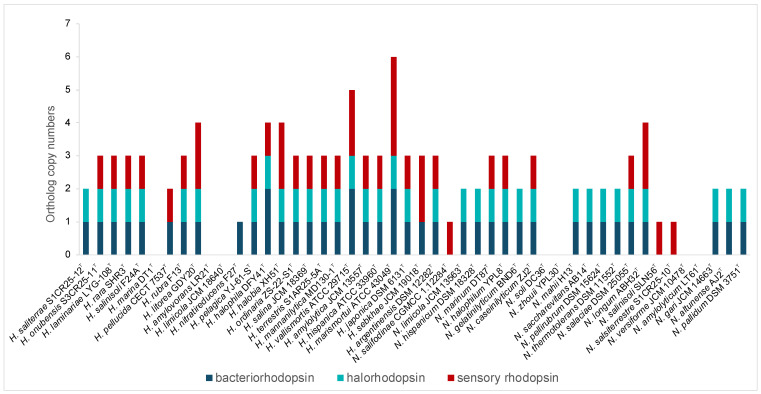
Distribution of genes encoding bacteriorhodopsin, halorhodopsin, and sensory rhodopsin in species of the genera *Haloarcula* and *Natrinema*.

In addition, rhodopsins, light-driven proteins critical for phototrophic growth, osmoregulation, and environmental sensing, were highly represented in both genera, especially *Haloarcula* (Figure 3, Supplementary Table S2). Bacteriorhodopsin, a light-driven proton pump, facilitates energy generation, while halorhodopsins help maintain osmotic balance by importing chloride ions. Sensory rhodopsins enable phototaxis, guiding movement toward or away from light sources [46]. The presence of the rhodopsins highlights the capability of these haloarchaea to harness light energy, supporting survival in hypersaline habitats and offering potential for various biotechnological applications, including optogenetics, bioelectronics, and bioenergy [47,48].

### 3.3. Osmoregulatory Mechanisms and Proteomic Adaptations

#### 3.3.1. “Salt-In” Strategy

**Figure 4:**
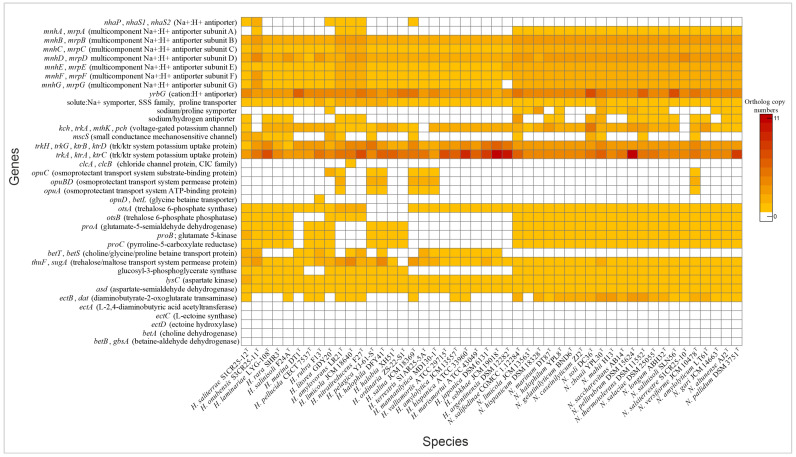
Heatmap of genes associated with “salt-in” and “salt-out” osmoregulatory mechanisms in *Haloarcula* and *Natrinema* species.

Haloarchaea employ a variety of mechanisms to maintain cellular homeostasis in response to fluctuating osmotic conditions. A key strategy involves the regulation of ion transporters, which control the movement of ions across the cell membrane. By adjusting intracellular ion concentrations, haloarchaea mitigate osmotic stress and maintain optimal cellular function. Both *Natrinema* and *Haloarcula* possessed specialized transporters that mediate K^+^ uptake and Na^+^ expulsion, crucial components of the “salt-in” strategy for maintaining osmotic balance in hypersaline environments (Figure 4). Additionally, Cl^−^ transporters, including chloride channels and halorhodopsins, play complementary roles. While chloride channels passively regulate chloride influx to maintain osmotic balance, halorhodopsins actively pump chloride ions into the cytoplasm using light energy, contributing to energy production [49,50]. Halorhodopsins were found in the majority of species from both genera (Figure 3), while a chloride channel was identified exclusively in *Haloarcula limicola* JCM 18640^T^ (Supplementary Table S2). To cope with hypoosmotic shock, small-conductance mechanosensitive channels (MscS) release ions and small compatible solutes to prevent cell lysis [51,52]. The *mscS* genes involved in this mechanism were identified in some species of *Natrinema* and *Haloarcula* (Figure 4, Supplementary Table S2), allowing them to survive sudden decreases in salinity.

#### 3.3.2. “Salt-Out” Strategy

Another key adaptation to osmotic stress is the accumulation of compatible solutes—part of the “salt-out” strategy—which stabilizes proteins and maintains cellular function without interfering with metabolic processes [53]. Our analysis showed that both genera contained genes for transport and *de novo* synthesis of compatible solutes. Previous research has shown that the organic solutes trehalose and glycine betaine are universally present in extremely halophilic archaea, obtained either through *de novo* biosynthesis or uptake from external sources [54]. Our genomic analysis revealed transport systems for a variety of solutes, including proline, choline, glycine betaine, proline betaine, and trehalose (Figure 4, Supplementary Table S2). The trehalose biosynthesis pathway (OtsAB) was detected in all *Natrinema* species, although it was less prevalent in *Haloarcula*. Conversely, a trehalose transporter was present in all *Natrinema* and *Haloarcula* species except *Haloarcula ordinaria* ZS-22-S1^T^. The proline biosynthesis pathway from glutamate was identified in all *Natrinema* species and in twelve *Haloarcula* species [55]. The *opuABC* gene cluster—which encodes an ABC transport system involved in the uptake of glycine betaine, proline betaine, and other small organic molecules—was present in *Natrinema versiforme* JCM 10478^T^ and several *Haloarcula* species, including *H. amylovorans* LR21^T^, *H. pelagica* YJ-61-S^T^, *H. halophila* DFY41^T^, *H. salina* JCM 18369^T^, *H. terrestris* S1AR25-5A^T^, and *H. mannanilytica* MD130-1^T^ (Figure 4, Supplementary Table S2). The biosynthesis pathway of glycine betaine from choline, mediated by BetAB [56], was not identified in any of the *Haloarcula* or *Natrinema* species. Similarly, the *de novo* ectoine biosynthesis pathway appeared incomplete. Aspartate kinase and aspartate semialdehyde dehydrogenase, which are involved in the initial steps of ectoine production, were present in all the studied genomes. However, these enzymes also participate in the formation of threonine, methionine, and isoleucine [57]. The *ectB* gene was detected in ten *Haloarcula* species and all *Natrinema* species, with multiple copies found in most *Natrinema* genomes. In contrast, the *ectA* and *ectC* genes were absent in all the studied genomes, along with *ectD*, which is responsible for converting ectoine into 5-hydroxyectoine, often considered a superior compatible solute due to its enhanced hydration and antioxidant properties [58,59]. In terms of glucosylglycerate biosynthesis, all the *Natrinema* species and approximately half of the *Haloarcula* species carried the *gpgS* gene (Figure 4, Supplementary Table S2) which encodes glucosyl-3-phosphoglycerate synthase, responsible for the first step of the pathway. However, none of the species contained the *gpgP* gene, encoding glucosyl-3-phosphoglycerate phosphatase, which converts glucosyl-3-phosphoglycerate to glucosylglycerate, another compatible solute [60]. This absence suggests the potential use of alternative enzymes, non-specific phosphatases, or reliance on other osmoregulatory mechanisms. Comprehensive genomic analysis suggests that *Haloarcula* and *Natrinema* demonstrate adaptability in osmoregulatory mechanisms, likely employing both “salt-in” and “salt-out” strategies in response to varying osmotic stress levels and resource availability.

#### 3.3.3. Proteome and Genomic Adaptation

**Figure 5:**
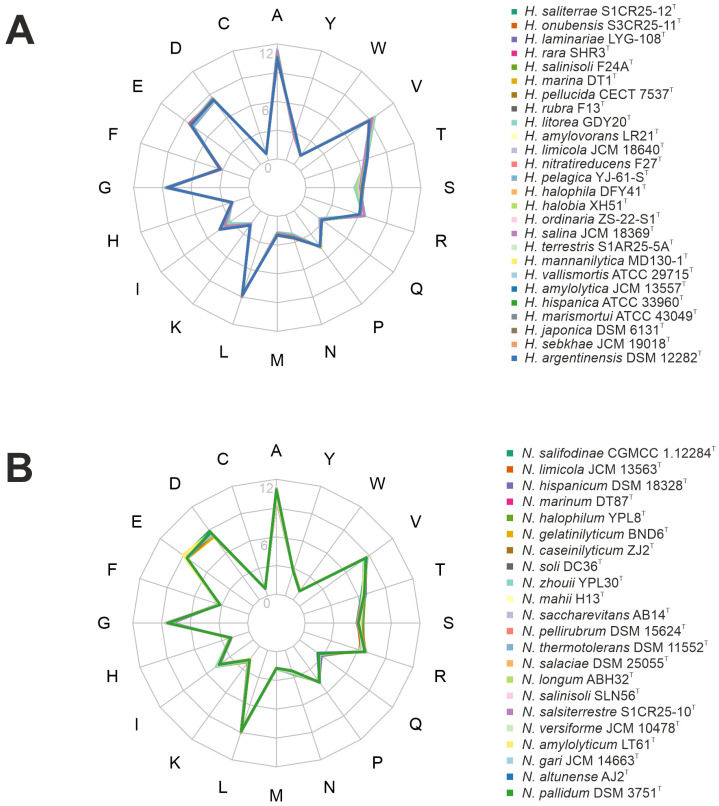
Amino acid frequency of the species of the genera (**A**) *Haloarcula* and (**B**) *Natrinema*.

Adaptations of haloarchaea to high-salt environments further involve both acidification of their proteome and an increased genomic G+C content [61]. Proteome acidification is achieved through a higher proportion of acidic amino acids, particularly aspartate (D) and glutamate (E). These acidic residues contribute to the surface charge of proteins, enhancing water retention and counteracting the destabilizing effects of high ionic concentrations. This adaptation ensures proper protein folding and functionality under extreme salinity, preventing aggregation and maintaining stability under osmotic stress [9]. In the studied genomes, aspartate and glutamate were prominently represented in both genera, particularly in *Natrinema* species, following alanine and leucine as the most abundant amino acids (Figure 5). Furthermore, both genera exhibited a high genomic G+C content, ranging from 60.1 to 65.9 mol%. While high G+C content is primarily associated with thermophilic adaptation, it also contributes to genomic stability in haloarchaea, which often face elevated temperatures and intense solar radiation in hypersaline environments. Additionally, G+C content can indirectly influence protein stability by shaping codon usage and amino acid composition [62]. Proteins encoded by G+C-rich genes may exhibit a higher prevalence of specific amino acids that enhance structural stability, aiding survival in high-salt conditions.

**Figure 6:**
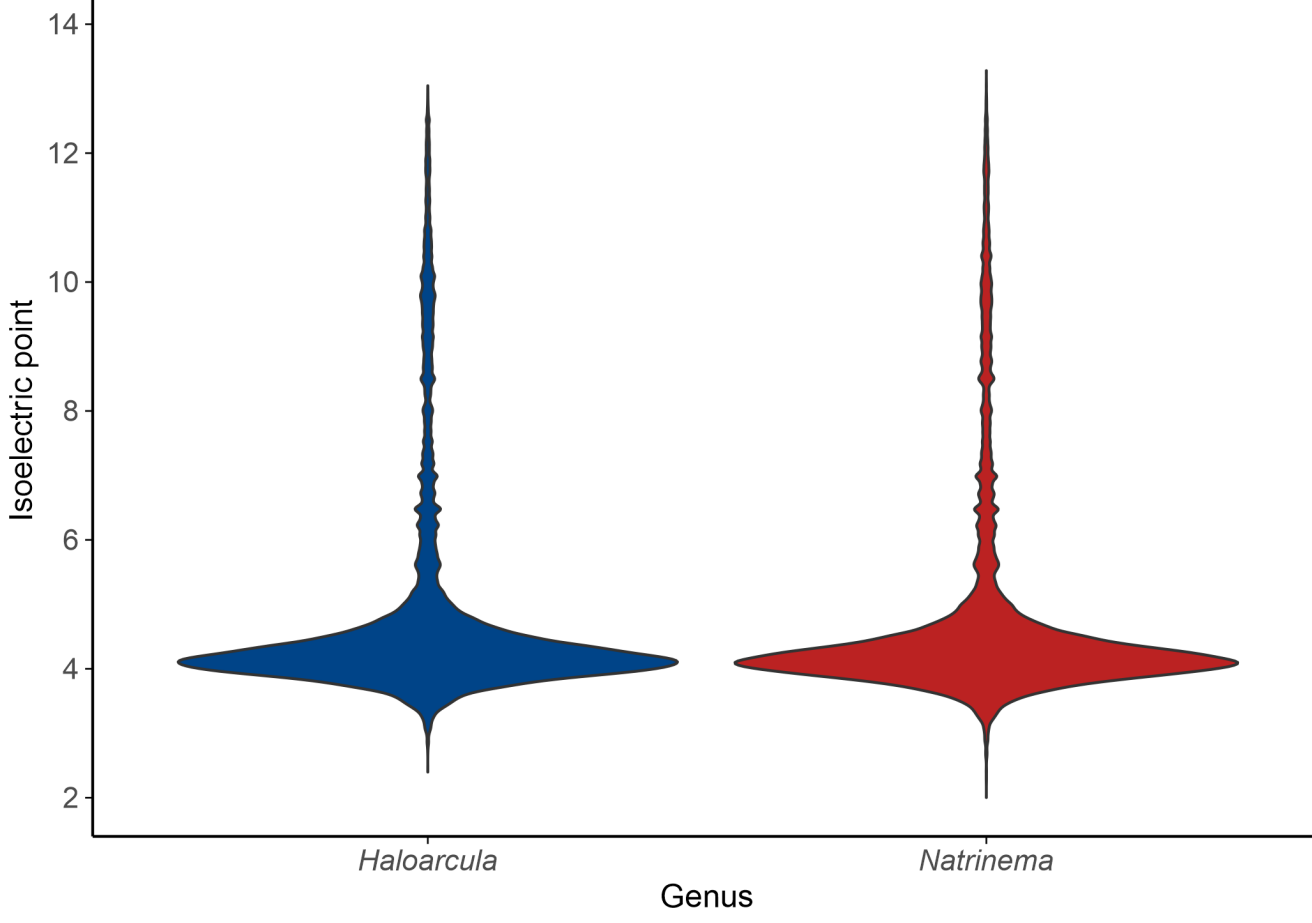
Isoelectric point distribution of predicted proteins in the genera *Haloarcula* and *Natrinema*.

The acid–base balance of the proteome is further visualized by the isoelectric point (pI) distribution. A shift towards acidic pI values (~4) indicates a predominance of acidic residues, consistent with the “salt-in” strategy for osmoregulation, while higher pI values (above 7) correspond to basic proteins [61]. The isoelectric point profiles of species of both *Haloarcula* and *Natrinema* revealed a predominantly acidic proteome, with major peaks around pI 4 (Figure 6); however, the extended distribution toward higher pI values indicates the presence of basic proteins, such as DNA-binding proteins, transporters, and membrane-associated proteins, which require a positive charge for proper function or interaction with cellular components. In summary, the bimodal distribution of isoelectric points reflects a balance between the adaptation of haloarchaea to hypersaline environments via proteome acidification and the functional necessity for basic proteins in specific cellular processes.

### 3.4. Mechanisms of Stress Tolerance

#### 3.4.1. Adaptation to High UV Radiation

Haloarchaea, a group of extremophilic archaea, have evolved to thrive in some of the most hostile environments, including hypersaline lakes, salterns, and evaporation ponds. These environments subject them to extreme conditions including not only high salinity, but also intense ultraviolet (UV) radiation and oxidative stress. Additionally, they must withstand fluctuating temperatures and periods of desiccation, posing significant challenges to their survival [1,63].

High levels of UV radiation present a significant threat to haloarchaea due to the potential DNA damage, including the formation of cyclobutane pyrimidine dimers (CPDs) and other DNA lesions. To protect against UV damage, haloarchaea produce carotenoid pigments such as bacterioruberin and β-carotene. These pigments absorb UV light and quench reactive oxygen species (ROS), preventing oxidative damage to cellular components [63]. The bacterioruberin synthesis pathway, involving enzymes such as LyeJ, CrtD, and CruF, was present in all studied species, and CrtY—involved in the production of β-carotene—was also identified in the majority of the species of both genera investigated (Supplementary Table S2). In addition to pigment-based photoprotection, haloarchaea utilize several DNA repair mechanisms. Photoreactivation, mediated by photolyase enzymes encoded by genes such as *phr* (K01669) and *phrB* (K06876), is one of the primary mechanisms for repairing UV-induced CPDs and 6–4 photoproducts, respectively [63]. These genes were present in both studied genera; however, only one *Natrinema* species possessed *phrB*, while it was more abundant in *Haloarcula* species (Supplementary Table S2). Another key repair mechanism is the nucleotide excision repair (NER) pathway, which removes a broad range of DNA lesions, including UV-induced damage. The genes *uvrA*, *uvrB*, *uvrC*, and *uvrD* encode the proteins responsible for recognizing and excising damaged DNA, followed by repair synthesis [64,65]. The DNA repair protein RadA further assists in homologous recombination repair, allowing for the accurate fix of double-strand breaks caused by UV radiation. RadB, in addition, may serve a supporting role in DNA repair mechanisms [66]. Base excision repair (BER) is another DNA repair mechanism in haloarchaea for addressing damage to individual bases caused by factors like oxidation, alkylation, or deamination. It consists of DNA glycosylases, apurinic/apyrimidinic (AP) endonucleases, DNA polymerases, and DNA ligases [67]. There is generally more variation in DNA glycosylases because they are responsible for recognizing a wide variety of different types of DNA lesions, and therefore, multiple specialized glycosylases have evolved to identify specific types of base damage, such as AlkA (alkyladenine glycosylase), MutY (A/G-specific adenine glycosylase), Nth (endonuclease III), and OggI (8-oxoguanine glycosylase I) [68]. The NER, BER, and homologous recombination repair mechanisms were present in all the studied species (Supplementary Table S2).

#### 3.4.2. Defense Against Oxidative Stress

In addition to UV radiation, haloarchaea are also exposed to high oxygen levels, which can lead to the formation of ROS and cause oxidative damage to biomolecules, including lipids, proteins, and DNA. To counteract oxidative stress, haloarchaea have evolved an antioxidant defense system involving both enzymatic and non-enzymatic components. Enzymatically, superoxide dismutase (SOD), particularly the Fe-Mn family (K04564), plays a critical role in neutralizing superoxide radicals (O_2_^•−^) by converting them into hydrogen peroxide (H_2_O_2_), which is less toxic and can be further broken down by catalase-peroxidase (K03782) into water and oxygen [64,69]. These enzymes were identified in all studied species. Peroxiredoxins, such as thioredoxin-dependent peroxiredoxin (K03564) and glutaredoxin-dependent peroxiredoxin (K24129), were also found across all the studied species (Supplementary Table S2). They play a vital role in reducing hydrogen peroxide and organic peroxides, protecting cellular components from oxidative damage. Moreover, both studied genera rely on methionine sulfoxide reductases (Msr) to repair oxidized methionine residues in proteins (Supplementary Table S2). The thioredoxin (TrxA, K03671) and thioredoxin reductase (TrxB, K00384) systems also maintain protein redox homeostasis by facilitating the reduction of disulfide bonds, thus preventing protein aggregation under oxidative stress [70,71]. They were present in all analyzed genomes. Glutaredoxins, particularly monothiol glutaredoxin (GrxD, K07390), found in all the studied species (Supplementary Table S2), play a role in protecting against oxidative stress by regulating iron–sulfur cluster metabolism and maintaining redox balance. The flavin-binding protein dodecin present in all the studied genomes (Supplementary Table S2) provides additional protection against oxidative damage [72]. Non-enzymatic antioxidants, such as carotenoids, further complement the oxidative defense system. Bacterioruberin and β-carotene (synthesized by the vast majority of analyzed species of *Haloarcula* and *Natrinema*, as aforementioned) can also neutralize ROS generated by oxidative stress, including those formed after UV radiation exposure. Due to their strong antioxidant properties, these pigments have garnered significant interest in biotechnological applications, particularly in the pharmaceutical and cosmetic industries, where they are explored for their potential in skin protection, anti-aging treatments, and as natural colorants [73]. Additionally, the *dps* gene present across species of both studied genera encodes a starvation-inducible DNA-binding protein that protects DNA from oxidative damage by binding to it, effectively shielding it from direct ROS interaction [74]. Similarly, the *bfr* gene encoding bacterioferritin sequesters iron and prevents the Fenton reaction, a process that generates highly reactive hydroxyl radicals from hydrogen peroxide [75]. This gene was present in all *Natrinema* species, but only in about half of *Haloarcula* species, especially those originally classified under the *Halomicroarcula* genus.

#### 3.4.3. Response to Temperature Fluctuations

Haloarchaea are regularly exposed to extreme temperature shifts in their natural habitats, particularly in environments such as salt flats, where temperatures can vary significantly from day to night and from season to season. To cope with these fluctuations and preserve cellular stability, they rely on a range of heat-shock proteins, such as chaperones and chaperonins [9]. DnaK (Hsp70), DnaJ (Hsp40), and GrpE constitute an ATP-dependent chaperone complex that plays a critical role in maintaining protein homeostasis by preventing aggregation and promoting the refolding of partially denatured proteins [76,77]. This complex was encoded by *Haloarcula* species; however, most *Natrinema* species lacked GrpE (Supplementary Table S2). In addition, archaeal chaperonins such as CCT (chaperonin-containing TCP-1) form large complexes that encapsulate misfolded proteins, providing a controlled environment that facilitates correct protein folding [78]. The genomic analysis has revealed the presence of multiple *cct* genes in the studied genomes (Supplementary Table S2). Prefoldins (PfdA, K04797; PfdB, K04798), identified in all analyzed genomes, also contribute to protein stabilization by delivering unfolded proteins to chaperonins [79]. All the studied genomes contained additional key proteins involved in the stress response, including small heat-shock proteins, such as HSP20 (K13993), which prevent protein aggregation by acting as a molecular buffer under stress conditions [80]. The studied haloarchaea also possessed genes encoding peptidyl-prolyl *cis*-*trans* isomerases (PPIases), including PPIA, PPIB (only *Haloarcula* species), and SlyD, which accelerate the folding of proline-containing proteins by catalyzing the *cis*-*trans* isomerization of peptide bonds [81,82]. Additionally, the cold-shock proteins, including CspA present in all the studied genomes, can act as RNA chaperones that prevent the formation of secondary structures in mRNA, facilitating efficient translation at low temperatures [83,84]. In addition, CcmG/DsbE (cytochrome c biogenesis protein), serine protease, thermitase, TorD, DmsD, hexosaminidase, HypAB, and small multidrug resistance pump, found in a minority of the studied species, further provide environmental adaptations (Supplementary Table S2).

#### 3.4.4. Protection Against Genetic and Environmental Threats

Haloarchaea have evolved a variety of intricate defense mechanisms to protect their genomes and cellular integrity against environmental threats such as viruses, plasmids, and harmful chemicals. Among these, the CRISPR-Cas system is an adaptive immune defense, allowing haloarchaea to recognize and target foreign genetic material [85]. CRISPR (clustered regularly interspaced short palindromic repeats) *loci* and Cas (CRISPR-associated) proteins were present in the genomes of several studied species, showing type I-B *cas* gene clusters, except for *Haloarcula saliterrae* S1CR25-12^T^, which contained the I-D *cas* type (Supplementary Tables S2 and S3). Furthermore, the studied species employ different defense strategies, such as the Restriction-Modification (R-M) and the Toxin-Antitoxin (TA) systems (Supplementary Table S2). *Haloarcula terrestris* S1AR25-5A^T^, in addition, possessed genes involved in the DNA phosphothiolation system, which modifies DNA by introducing sulfur atoms into the DNA backbone, specifically in the phosphate groups, which can protect the genome against various environmental threats, such as nucleases and oxidative agents [86]. Together, these defense mechanisms—CRISPR-Cas, R-M, TA, and DNA phosphothiolation systems—equip haloarchaea with a multi-faceted strategy to counteract genetic threats, regulate cellular stress, and maintain genomic stability, ensuring their survival in extreme environments.

### 3.5. Heavy Metal Tolerance Mechanisms in Haloarcula and Natrinema

#### 3.5.1. Arsenic Resistance Mechanisms

**Figure 7:**
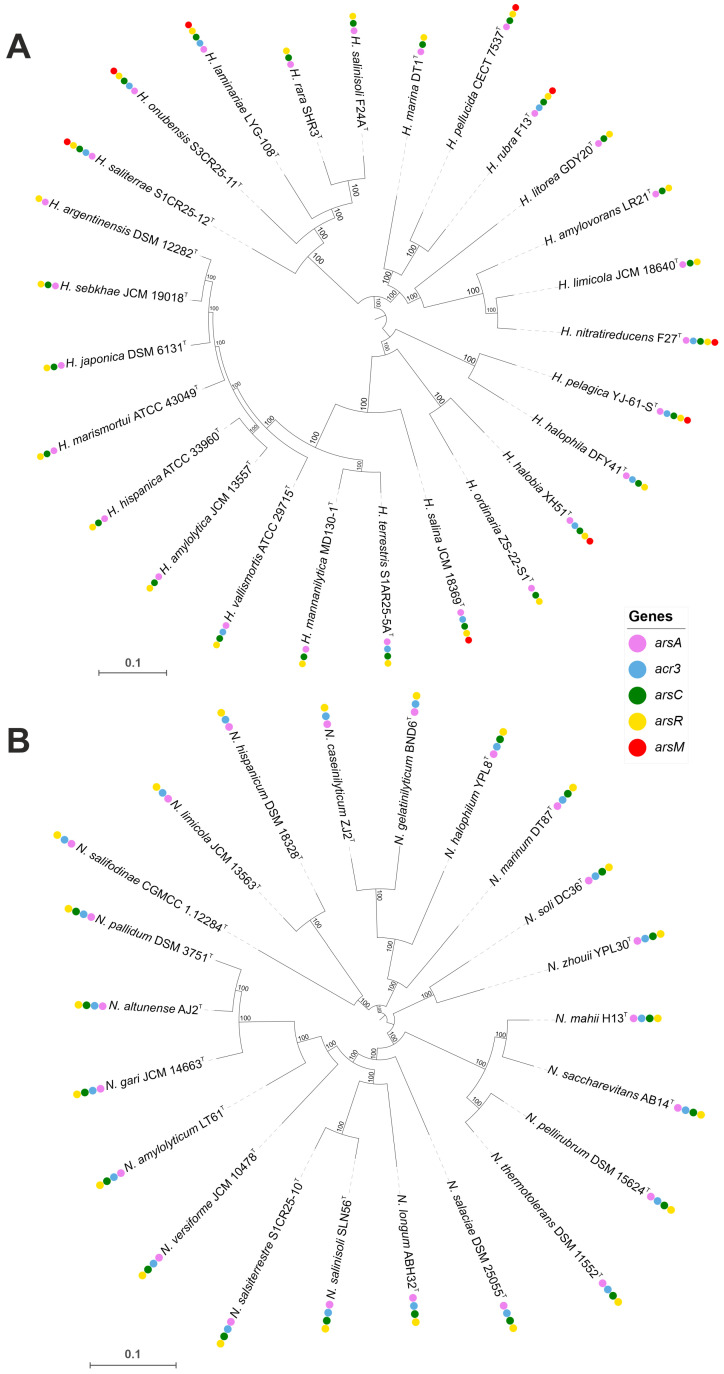
Approximate maximum-likelihood phylogenomic trees showing the distribution of arsenic resistance genes in *Haloarcula* (**A**) and *Natrinema* (**B**) species. The presence of specific resistance genes is mapped alongside species names, showing clustering patterns and gene conservation among closely related taxa. Bootstrap values are indicated at branch nodes. Bars, 0.1 substitutions per amino acid position.

**Figure 8:**
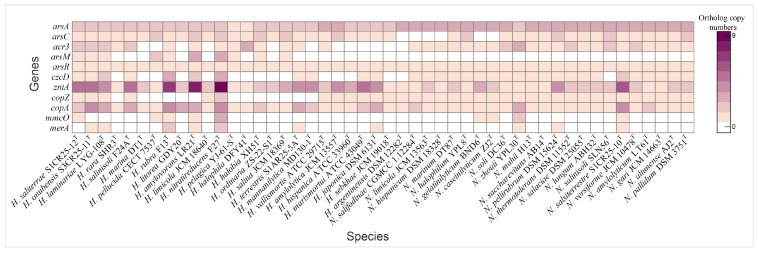
Heatmap illustrating the distribution of heavy metal tolerance genes in species of the genera *Haloarcula* and *Natrinema*.

Haloarchaea have developed a range of molecular mechanisms to tolerate and/or resist heavy metal toxicity, enabling their survival in hypersaline environments often contaminated with heavy metals. Arsenic resistance is mediated by a specialized set of proteins, including the arsenite transporter (*acr3*, K03325), which actively exports arsenite to prevent intracellular accumulation, and the arsenate reductase *arsC* (K03741), which reduces arsenate to arsenite for subsequent removal. The activity of Acr3 transporter is enhanced in the presence of ArsA, as demonstrated by Castillo and Saier [87]. Additional detoxification occurs via arsenite methylation by *arsM* (K07755), which converts arsenite into less toxic methylated derivatives. This arsenic detoxification system is regulated by the ArsR family transcriptional regulator (K07721), which activates arsenic resistance genes in response to arsenic exposure [88,89]. Comparative genomic analysis revealed that arsenic resistance genes are highly conserved across both genera; however, the *arsM* gene was detected only in certain *Haloarcula* species (Figure 7 and Figure 8, Supplementary Table S2).

#### 3.5.2. Copper Detoxification Pathways

Copper resistance mechanisms involve a copper chaperone (CopZ, K07213), which facilitates copper ion delivery to copper-dependent enzymes, and the P-type Cu^+^ transporter CopA, an ATPase responsible for exporting copper ions to mitigate toxicity [90,91]. Additional detoxification is achieved by MmcO (K22552), a multicopper oxidase that detoxifies copper ions by oxidation [92]. The copper chaperone was universally present in *Natrinema* species and in half of the *Haloarcula* species, while *copA* was found in all species, particularly those isolated from the Odiel Saltmarshes. The *mmcO* gene was identified in *H. saliterrae* S1CR25-12^T^, *H. rubra* F13^T^, *H. amylovorans* LR21^T^, *H. nitratireducens* F27^T^, *N. gelatinilyticum* BND6^T^, *N. caseinilyticum* ZJ2^T^, *N. zhouii* YPL30^T^, and *N. salsiterrestre* S1CR25-10^T^ (Figure 8, Supplementary Table S2).

#### 3.5.3. Zinc, Cadmium, and Cobalt Efflux Systems

Additional heavy metal resistance mechanisms include specific transporters and efflux systems, such as the cobalt-zinc-cadmium efflux protein CzcD (K16264) and the Zn^2^^+^/Cd^2^^+^-exporting ATPase ZntA (K01534), which prevent the toxic accumulation of metals by exporting excess ions [93,94]. A metal-responsive transcriptional regulator (K21903), which modulates the expression of efflux and detoxification genes in response to lead, cadmium, zinc, and bismuth, was only identified in *Natrinema salifodinaeae* CGMCC 1.12284^T^ (Supplementary Table S2). While *czcD* was present in select *Haloarcula* species, especially those from the Odiel Saltmarshes, all *Natrinema* species except *Natrinema salinisoli* CGMCC 1.12284^T^ contained this gene. The *zntA* gene was universally present in all the studied species, with the highest copy number found in isolates from the Odiel Saltmarshes (Figure 8, Supplementary Table S2). The *merA* gene, encoding mercuric reductase, was present in some studied genomes (Figure 8, Supplementary Table S2). These findings highlight the genomic versatility of *Haloarcula* and *Natrinema* species in coping with heavy metal stress, especially within the challenging conditions of hypersaline environments such as the Odiel Saltmarshes.

#### 3.5.4. Determination of Minimum Inhibitory Concentrations (MICs) for Heavy Metal Tolerance

Based on this in silico functional genomic analysis and prior research revealing elevated concentrations of heavy metals, such as arsenic, cadmium, copper, lead, and zinc in the hypersaline soils of the Odiel Saltmarshes [95,96,97], three *Haloarcula* species and one *Natrinema* species previously isolated from this region were selected for experimental validation of their heavy metal tolerance. Minimal inhibitory concentration (MIC) assays were conducted to assess the tolerance of the four selected species to these heavy metals. The experimental results (Table 1) revealed significant heavy metal tolerance among the studied haloarchaeal species, reinforcing the evidence that haloarchaea have evolved exceptional adaptations to thrive in metal-rich environments. Their extreme tolerance to arsenic (up to 700 mM) surpasses previously documented levels in haloarchaea, such as those reported by Ordoñez et al. [98], who observed arsenic resistance up to 250 mM. This suggests that the studied strains may possess enhanced arsenic resistance mechanisms. Similarly, their substantial cadmium tolerance aligns with the findings of Tavoosi et al. [99], who reported comparable resistance levels in two *Natrinema* strains, one *Haloarcula* strain, and one *Halococcus* strain. The ability to grow in the presence of copper varied among species. *Haloarcula terrestris* S1AR25-5A^T^ and *Haloarcula saliterrae* S1CR25-12^T^ tolerated copper concentrations of 2.5 mM and 4 mM, respectively, while *Haloarcula onubensis* S3CR25-11^T^ and *Natrinema salsiterrestre* S1CR25-10^T^ demonstrated slight growth even at copper concentrations of 10 mM. The highest copper tolerance in halophilic archaea reported to date is 32 mM in *Halovarius luteus* DA5 [99]. Lead tolerance appeared to be a conserved trait across the studied haloarchaeal species, as all strains demonstrated the ability to grow in the presence of 5 mM lead. This is consistent with previous findings by Nieto et al. [31], who reported that all the tested haloarchaeal strains exhibited lead resistance, with many tolerating up to 10 mM and some enduring even higher concentrations. Zinc tolerance is generally lower, with reported concentrations not exceeding 1–2 mM [99,100]. In this study, the lowest MIC values were also attributed to zinc, with concentrations not surpassing 1 mM. Overall, the results obtained in this study closely align with previous research on species isolated from the Odiel Saltmarshes Natural Area [97], as well as from other hypersaline environments, including the Andes [98] and Iran [99], further substantiating the resilience of haloarchaea in heavy metal-rich environments. Additionally, salinity can significantly affect heavy metal resistance in halophilic microorganisms, with moderate salinity enhancing metal uptake and resistance, while excessively high salinity can inhibit these processes [101,102]. Understanding these dynamics is essential for developing bioremediation strategies in hypersaline environments.

### 3.6. Functional Analysis of Thiamine Biosynthesis Pathway

The biosynthesis of thiamine (vitamin B_1_) is a critical metabolic pathway responsible for producing thiamine diphosphate (ThDP), a coenzyme involved in key enzymatic reactions of carbohydrate metabolism and amino acid biosynthesis. *De novo* thiamine synthesis in the studied haloarchaea follows the two-branch pathway observed in other organisms, where separate branches produce the pyrimidine and thiazole moieties. Hydroxymethylpyrimidine pyrophosphate (HMP-PP) is synthesized from aminoimidazole ribotide (AIR), a purine biosynthesis intermediate, through the catalytic activity of the enzymes ThiC and ThiD/ThiDN [103,104]. The formation of the thiazole ring in archaea involves the activity of Thi4, a eukaryotic-like protein homolog, along with a presumed NUDIX hydrolase. Once synthesized, the thiamine ring precursors, 4-methyl-5-[β-hydroxyethyl] thiazole phosphate (THZ-P) and HMP-PP, are combined to form thiamine monophosphate (ThMP) through the catalytic action of a ThMP synthase, either of the ThiE or ThiN type [105,106,107]. ThMP is subsequently phosphorylated by ThiL to produce the biologically active coenzyme, ThDP [108].

**Figure 9:**
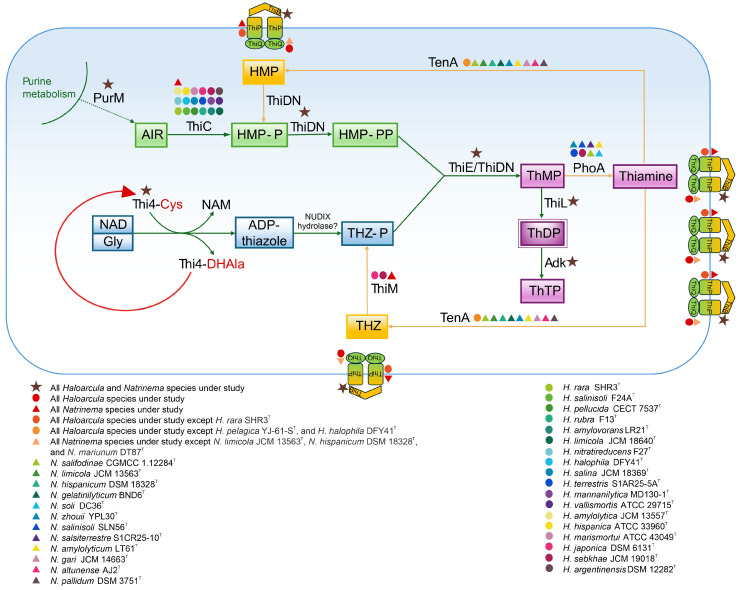
Biosynthesis and salvage pathways of thiamine (vitamin B_1_) in *Haloarcula* and *Natrinema* species based on functional genomic analysis. The presence of enzymes in the studied *Haloarcula* and *Natrinema* species is represented by colored symbols, with designations provided in the figure legend. ADP-thiazole, ADP-5-ethyl-4-methylthiazole-2-carboxylate; AIR, 5′-phosphoribosyl-5-aminoimidazole; Cys, cysteine; DHAla, dehydroalanine; Gly, glycine; HMP, 4-amino-5-hydroxymethyl-2-methylpyrimidine; HMP-P, 4-amino-5-hydroxymethyl-2-methylpyrimidine phosphate; HMP-PP, 4-amino-5-hydroxymethyl-2-methylpyrimidine diphosphate; NAD, nicotinamide adenine dinucleotide; NAM, nicotinamide; ThMP, thiamine monophosphate; ThDP, thiamine diphosphate; ThTP, thiamine triphosphate; THZ, 4-methyl-5-(β-hydroxyethyl) thiazole; THZ-P, 4-methyl-5-(β-hydroxyethyl) thiazole phosphate. The enzymes are discussed in the text.

The functional genomic analysis revealed that the *thiC* gene, encoding the enzyme responsible for the initial step in aminopyrimidine moiety synthesis (Figure 9), was present in all *Natrinema* species and in the majority of *Haloarcula* members (Supplementary Table S2). The absence of *thiC* in some *Haloarcula* species results in an incomplete thiamine biosynthetic pathway. In contrast, the *thiDN* gene was identified in all the studied species. Haloarchaea possess Thi4 homologs containing a conserved active-site cysteine residue, which is essential for the synthesis of adenylated thiazole (ADT) through a single-turnover reaction involving the conversion of nicotinamide adenine dinucleotide (NAD) and glycine [109]. These functional homologs were detected across all the species under study (Supplementary Tables S2 and S4). The formation of ThMP is catalyzed by either ThiE or ThiN, with the ThiN domain often occurring as part of the fusion protein ThiDN. The archaeal ThiDN protein is a multifunctional enzyme capable of catalyzing three consecutive steps in the *de novo* synthesis of vitamin B_1_ [105] (Figure 9). Interestingly, certain extremophilic microorganisms, such as *Pyrococcus furiosus* and *Sulfolobus solfataricus*, possess both *thiDN* and *thiE* genes, indicating coexisting pathways for thiamine monophosphate synthesis [105]. In the current study, both *thiDN* and *thiE* genes were detected in all the analyzed species, suggesting evolutionary advantages such as enhanced metabolic flexibility and adaptation to fluctuating environmental conditions (Supplementary Table S2). The enzyme ThiL, responsible for catalyzing the final step in ThDP formation, was found in all the studied haloarchaea. Additionally, adenylate kinase (Adk) facilitates the phosphorylation of ThDP to produce thiamine triphosphate (ThTP) (Figure 9). ThTP, a multifunctional molecule associated with energy metabolism, stress response, and cellular signaling [110,111], however, remains poorly understood in archaea. ThiR, identified in all species of both genera, is a central regulatory protein ensuring balanced thiamine biosynthesis and transport depending on environmental and intracellular conditions [104,106]. Taken together, the findings indicate that all the studied *Natrinema* species and the majority of *Haloarcula* species possessing the *thiC* gene have the potential for *de novo* thiamine biosynthesis.

In addition to the biosynthetic pathway, the thiamine salvage pathway plays a critical role in minimizing energy consumption and preventing the incorporation of thiamine degradation products into ThDP-dependent enzymes. This pathway is anticipated to involve enzymes from *de novo* synthesis (ThiDN, ThiE, and ThiL) alongside salvage-specific enzymes such as ThiM and TenA [107]. The *thiM* gene, responsible for the phosphorylation of 4-methyl-5-(β-hydroxyethyl) thiazole (THZ), was detected in all *Natrinema* species but only in two *Haloarcula* members (Supplementary Table S2). This absence in most *Haloarcula* species might indicate a reliance on an external thiamine supply or *de novo* synthesis. In contrast, the *tenA* gene, involved in salvaging base-degraded thiamine derivatives [107,112], was more prevalent in *Haloarcula* species than in *Natrinema* representatives (Supplementary Table S2). Additionally, alkaline phosphatase (PhoA), which supports thiamine recycling and the salvage pathway, was detected in only a few species, predominantly within the genus *Natrinema*. The transport of thiamine, its phosphorylated derivatives, and intermediates is facilitated by the thiamine transport system, comprising ThiB (K02064), ThiP (K02063), and ThiQ (K02062) subunits [113] (Figure 9), ensuring adaptability to environmental nutrient availability. The *thiB* gene was present in all *Haloarcula* and *Natrinema* species; while *thiP* was not annotated for *H. rara* SHR3^T^; and *thiQ* was absent in *N. limicola* JCM 13563^T^, *N. hispanicum* DSM 18328^T^, and *N. marinum* DT87^T^ (Supplementary Table S2).

### 3.7. Research Implications

This study provides valuable insights into stress adaptation and metabolic versatility in *Haloarcula* and *Natrinema*, while also emphasizing their potential for various biotechnological applications. The characterization of heavy metal resistance mechanisms, particularly the exceptional arsenic tolerance observed in some species, with MIC values reaching up to 700 mM, highlights the potential of these haloarchaea for the bioremediation of arsenic-contaminated environments. These microorganisms also show promise for the production of valuable biomolecules, offering sustainable alternatives to traditional methods. For instance, they harbor genes involved in the biosynthesis of polyhydroxyalkanoates (PHAs), a class of biodegradable and biocompatible biopolymers with thermoplastic properties. PHAs have gained attention as sustainable alternatives to conventional plastics, with potential applications in packaging, medical implants, and drug delivery systems [39,40]. While challenges remain in optimizing PHA production at an industrial scale, haloarchaea offer advantages due to their ability to grow in high-salinity conditions, reducing the risk of contamination. Furthermore, the presence of rhodopsin-related genes in the studied *Haloarcula* and *Natrinema* species highlights their potential in optogenetics, bioelectronics, and bioenergy [47,48]. Carotenoids such as bacterioruberin and β-carotene, identified in the studied species through functional genomic analysis, have broad applications in the food and cosmetic industries, as well as in biomedicine, where they serve as antioxidants, anti-tumor agents, cardiovascular protectants, and vitamin A precursors [114]. Furthermore, the discovery of *de novo* thiamine synthesis in most of the studied species presents potential applications in biotechnology. Thiamine is essential for supporting the nervous and cardiovascular systems, strengthening the immune response, and enhancing cognitive functions [115]. It has potential in drug development, particularly in targeting cancer and fungal infections [116]. While vitamin B_1_ is predominantly produced through chemical synthesis on an industrial scale, elucidating its biosynthetic pathway in haloarchaea may enable future biotechnological production. Additionally, thiamine plays a role in nutrient cycling and could contribute to improving crop resistance [107]. Finally, the present work provides a foundation for future studies, integrating transcriptomics and proteomics approaches to validate the genomic predictions and further elucidate the regulatory networks underlying these adaptive mechanisms. Gaining deeper insights into these processes will not only expand our fundamental understanding of haloarchaeal physiology but also contribute to the development of sustainable biotechnological applications.

## 4. Conclusions

This study provides a comprehensive analysis of the environmental adaptation strategies in the haloarchaeal genera *Haloarcula* and *Natrinema*, focusing on their genomic diversity, metabolic capabilities, and mechanisms for stress resistance. The findings reveal adaptive mechanisms, such as DNA repair systems, antioxidant and immune defenses, “salt-in” and “salt-out” osmoregulatory strategies, protection against thermal shifts, and heavy metal detoxification pathways, which enable these extremophiles to thrive under poly-extreme conditions. Comparative genomic analysis highlights their metabolic versatility, including pathways for phototrophy, nitrogen assimilation, and thiamine biosynthesis, which demonstrate their ecological resilience and potential for biotechnological applications. In particular, the elucidation of heavy metal resistance mechanisms offers a valuable basis for exploring haloarchaea in sustainable bioremediation strategies for heavy metal-contaminated environments. The identification of genes encoding efflux transporters, reductases, regulatory proteins, and other components of heavy metal resistance pathways underscores the genomic plasticity and adaptability of the *Haloarcula* and *Natrinema* genera. Experimental validation of four representative species confirmed their exceptional heavy metal tolerance, especially to arsenic. These findings provide a foundation for future research on haloarchaeal physiology and their optimization for potential biotechnological applications. Integrating transcriptomics and proteomics approaches to validate these genomic predictions can further elucidate the regulatory networks governing these adaptive mechanisms. Furthermore, this knowledge may contribute to the development of sustainable strategies to address environmental challenges, including heavy metal contamination, plastic pollution, increasing salinization, and the need for sustainable production of valuable biomolecules.

## Figures and Tables

**Table 1:** Minimum inhibitory concentrations (MICs) of heavy metals in four haloarchaeal species. Heavy metal concentrations tested ranged from 0.01 mM to a maximum of 700 mM. Boldface values indicate the maximum tested concentrations where tolerance to the corresponding metal ion was observed, suggesting that the actual MICs exceed these values for certain species.

Species	MIC (mM) of:				
	As^5+^	Cd^2+^	Cu^2+^	Pb^2+^	Zn^2+^
*Haloarcula terrestris* S1AR25-5A^T^	**>700**	80	4	10	1
*Haloarcula saliterrae* S1CR25-12^T^	**>700**	10	5	10	1
*Haloarcula onubensis* S3CR25-11^T^	500	50	20	10	1
*Natrinema salsiterrestre* S1CR25-10^T^	**>700**	50	20	10	1

## Data Availability

The data presented in this study are openly available in GenBank/EMBL/DDBJ databases at https://www.ncbi.nlm.nih.gov/genbank/ (accessed on 14 August 2024), reference numbers included in the Supplementary Materials.

## Supplementary Materials

The following supporting information can be downloaded at: https://www.mdpi.com/article/10.3390/microorganisms13040761/s1, Table S1: Genome accession numbers of species of *Haloarcula* and *Natrinema* used in the study; Table S2: Overview of KEGG Modules and KEGG Orthology (KO) numbers and their associated definitions as annotated within the genomes of *Haloarcula* (A) and *Natrinema* (B) species; Table S3: CRISPR-Cas system in species of the genera *Haloarcula* and *Natrinema*; Table S4: Alignment of the active-site region of protein sequences of Thi4 homologs encoded by *thi4* genes across species of the genera *Haloarcula* and *Natrinema*.
