# Supplementary material for: Strategies of Environmental Adaptation in the Haloarchaeal Genera Haloarcula and Natrinema
Source: Microorganisms. 2025 Mar 27;13(4):761. doi: 10.3390/microorganisms13040761 (PMC12029414; doi:10.3390/microorganisms13040761)
Supplement: Supplementary file 1 [file microorganisms-13-00761-s001.zip › Supplementary_Material.pdf]

Supplementary Table S1. Genome accession numbers of species of *Haloarcula* and *Natrinema* used in this study.

**Species**	**Accession number**
H. saliterrae S1CR25-12^T	JAMQON000000000
H. onubensis S3CR25-11^T	JAMQOS000000000
H. laminariae LYG-108^T	JAMZFY000000000
H. rara SHR3^T	CP119785–CP119786
H. salinisoli F24A^T	RKLQ00000000
H. marina DT1^T	CP100404
H. pellucida CECT 7537^T	RKLW00000000
H. rubra F13^T	RKLR00000000
H. litorea GDY20^T	CP119779–CP119781
H. amylovorans LR21^T	SRIF00000000
H. limicola JCM 18640^T	JAHQXF000000000
H. nitratireducens F27^T	RKLT00000000
H. pelagica YJ-61-S^T	CP126161–CP126164
H. halophila DFY41^T	CP119559–CP119562
H. halobia XH51^T	CP119787–CP119788
H. ordinaria ZS-22-S1^T	CP119789–CP119790
H. salina JCM 18369^T	JAHQXE000000000
H. terrestris S1AR25-5A^T	JAMQOM000000000
H. mannanilytica MD130-1^T	BIXZ00000000
H. vallismortis ATCC 29715^T	AOLQ00000000
H. amylolytica JCM 13557^T	AOLW00000000
H. hispanica ATCC 33960^T	CP002921
H. marismortui ATCC 43049^T	CP039138
H. japonica DSM 6131^T	AOLY00000000
H. sebkhae JCM 19018^T	BMPD00000000
H. argentinensis DSM 12282^T	AOLX00000000
N. salifodinae CGMCC 1.12284^T	FOIS00000000
N. limicola JCM 13563^T	AOIT00000000
N. hispanicum DSM 18328^T	SHMP00000000
N. marinum DT87^T	CP100763
N. halophilum YPL8^T	CP058601
N. gelatinilyticum BND6^T	CP100756
N. caseinilyticum ZJ2^T	CP100445
N. soli DC36^T	CP084472
N. zhouii YPL30^T	CP059154
N. mahii H13^T	JHUT00000000
N. saccharevitans AB14^T	LWLN00000000
N. pellirubrum DSM 15624^T	AOIE00000000
N. thermotolerans DSM 11552^T	AOIR00000000
N. salaciae DSM 25055^T	FOFD00000000
N. longum ABH32^T	CP071463
N. salinisoli SLN56^T	CP084469
N. salsiterrestre S1CR25-10^T	JAMQOT00000000
N. versiforme JCM 10478^T	AOID00000000
N. amylolyticum LT61^T	JAIWPJ000000000
N. gari JCM 14663^T	AOIJ00000000
N. altunense AJ2^T	JNCS00000000
N. pallidum DSM 3751^T	AOII00000000

Supplementary Table S3. CRISPR-Cas system in species of the genera *Haloarcula* and *Natrinema*. The table lists the total number of CRISPR *loci* and spacers, along with the associated Cas type for each species.

**Species**	**CRISPR *loci***	**Cas Type**	**Spacers**
H. saliterrae S1CR25-12^{T}	13	I-D	49
H. onubensis S3CR25-11^{T}	12	I-B	75
H. laminariae LYG-108^{T}	14	I-B	40
H. salinisoli F24A^{T}	10	I-B	103
H. rubra F13^{T}	7	I-B	105
H. nitratireducens F27^{T}	11	I-B	97
H. terrestris S1AR25-5A^{T}	9	I-B	82
H. mannanilytica MD130-1^{T}	8	I-B	85
H. hispanica ATCC 33960^{T}	4	I-B	17
H. argentinensis DSM 12282^{T}	5	I-B	24
N. salifodinae CGMCC 1.12284^{T}	4	I-B	10
N. gari JCM 14663^{T}	4	I-B	176
N. halophilum YPL8^{T}	6	I-B	84
N. mahii H13^{T}	11	I-B	68
N. saccharevitans AB14^{T}	5	I-B	134
N. thermotolerans DSM 11552^{T}	3	I-B	103
N. pallidum DSM 3751^{T}	7	I-B	43

Supplementary Table S4. Alignment of the active-site region of protein sequences of Thi4 homologs encoded by *thi4* genes across species of the genera *Haloarcula* and *Natrinema*. The conserved active-site cysteine residue, analogous to Cys165 in *Haloferax volcanii* DS2^{T} (locus_tag = HVO_0665), is highlighted in red.

**Species**	**Thi4 homologs**
H. saliterrae S1CR25-12^{T}	VHALPREITCVDPIAVESDI
H. onubensis S3CR25-11^{T}	VHALPREITCVDPIAVEADL
H. laminariae LYG-108^{T}	VHALPREITCVDPIAVESEL
H. rara SHR3^{T}	VHALPREITCVDPIAVESDI
H. salinisoli F24A^{T}	VHALPREITCVDPIAVESDI
H. marina DT1^{T}	VHALPREITCVDPIAVESDL
H. pellucida CECT 7537^{T}	VHALPREITCVDPIAVEADL
H. rubra F13^{T}	VHALPREITCVDPIAVEADL
H. litorea GDY20^{T}	VHALPREITCVDPIAVEADL
H. amylovorans LR21^{T}	VHALPREITCVDPIAVEADL
H. limicola JCM 18640^{T}	VHALPREITCVDPIAVEADL
H. nitratireducens F27^{T}	VHALPREITCVDPIAVEADL
H. pelagica YJ-61-S^{T}	VHALPREITCVDPIAVEADL
H. halophila DFY41^{T}	VHALPREITCVDPIAVEADL
H. halobia XH51^{T}	VHALPREITCVDPIAVESDL
H. ordinaria ZS-22-S1^{T}	VHALPREITCVDPIAVEADL
H. salina JCM 18369^{T}	VHALPREITCVDPIAVEADL
H. terrestris S1AR25-5A^{T}	VHALPREITCVDPIAVEADL
H. mannanilytica MD130-1^{T}	VHALPREITCVDPIAVEADL
H. vallismortis ATCC 29715^{T}	VHALPREITCVDPIAVEADL
H. amylolytica JCM 13557^{T}	VHALPREITCVDPIAVEADL
H. hispanica ATCC 33960^{T}	VHALPREITCVDPIAVEADL
H. marismortui ATCC 43049^{T}	VHALPREITCVDPIAVEADL
H. japonica DSM 6131^{T}	VHALPREITCVDPIAVEADL
H. sebkhae JCM 19018^{T}	VHALPREITCVDPIAVEADL
H. argentinensis DSM 12282^{T}	VHALPREITCVDPIAVEADL
N. salifodinae CGMCC 1.12284^{T}	VHALPREITCVDPIAVEADL
N. limicola JCM 13563^{T}	VHALPREITCVDPIAVEADL
N. hispanicum DSM 18328^{T}	VHALPREITCVDPIAVEADL
N. marinum DT87^{T}	VHALPREITCVDPIAVEADL
N. halophilum YPL8^{T}	VHALPREITCVDPIAVEADL
N. gelatinilyticum BND6^{T}	VHALPREITCVDPIAVEADL
N. caseinilyticum ZJ2^{T}	VHALPREITCVDPIAVEADL
N. soli DC36^{T}	VHALPREITCVDPIAVEADL
N. zhouii YPL30^{T}	VHALPREITCVDPIAVEADL
N. mahii H13^{T}	VHALPREITCVDPIAVEADL
N. saccharevitans AB14^{T}	VHALPREITCVDPIAVEADL
N. pellirubrum DSM 15624^{T}	VHALPREITCVDPIAVEADL
N. thermotolerans DSM 11552^{T}	VHALPREITCVDPIAVEADL
N. salaciae DSM 25055^{T}	VHALPREITCVDPIAVEADL
N. longum ABH32^{T}	VHALPREITCVDPIAVEADL
N. salinisoli SLN56^{T}	VHALPREITCVDPIAVEADL
N. salsiterrestre S1CR25-10^{T}	VHALPREITCVDPIAVEADL
N. versiforme JCM 10478^{T}	VHALPREITCVDPIAVEADL
N. amylolyticum LT61^{T}	VHALPREITCVDPIAVEADL
N. gari JCM 14663^{T}	VHALPREITCVDPIAVEADL
N. altunense AJ2^{T}	VHALPREITCVDPIAVEADL
N. pallidum DSM 3751^{T}	VHALPREITCVDPIAVEADL
Haloferax volcanii DS2^{T}	VHALPRELTCVDPIAVESDL
